# Can urinary CTX-II be a biomarker for knee osteoarthritis?

**DOI:** 10.1186/s42836-020-0024-2

**Published:** 2020-02-10

**Authors:** Piti Arunrukthavon, Danai Heebthamai, Prapasri Benchasiriluck, Supinda Chaluay, Thanainit Chotanaphuti, Saradej Khuangsirikul

**Affiliations:** 1 Department of Orthopedic Surgery, Phramongkutklao Hospital and College of Medicine, Bangkok, Thailand; 2 Department of Orthopedic Surgery, Queen Savang Vadhana Memorial Hospital, Sriracha, Chonburi, Thailand; 3 Center of Private Research and Innovation Accelerator, Phyathai 2 Hospital, Bangkok, Thailand; 4 Phyathai Orthopedic Institute, Phyathai 2 Hospital, Bangkok, Thailand

**Keywords:** Biomarker, Urinary CTX-II, Knee osteoarthritis

## Abstract

**Background:**

Early diagnosis of knee osteoarthritis (OA) remains a diagnostic challenge. Urinary C-terminal cross-linked telopeptide of type II collagen (urinary CTX-II) is one of the potential OA biomarkers. However, conclusive evidence regarding the use of this biomarker as a tool for early diagnosis is still lacking. The purposes of this study were to compare urinary CTX-II levels in patients with knee OA and in healthy controls, to evaluate the correlation between urinary CTX-II levels, radiographic severity of OA, and patient-reported outcomes and to evaluate the effect of age and gender on urinary CTX-II levels in the Asian populations.

**Methods:**

Two groups were studied. The OA group included 78 patients with knee OA aged > 40 years who met the diagnostic criteria for knee OA described by the American College of Rheumatology (ACR). The control group consisted of 51 healthy participants age > 40 years without clinical or radiographic evidence of knee OA. Bilateral knee radiographs were taken and classified according to the Kellgren and Lawrence (KL) grading system. Urinary CTX-II was measured using a competitive ELISA test and Western Ontario and Mcmaster Universities Arthritis Index (WOMAC) was also recorded in all participants.

**Results:**

Urinary CTX-II was significantly higher in the OA group than in the control group (*p* < 0.001). The severe knee OA group (KL grade 3 and 4) had higher urinary CTX-II levels than mild knee OA group (KL grade 2) but the difference did not reach statistical significance (*p* = 0.2). There was a moderate correlation between urinary CTX-II levels and KL grades (r = 0.405, *p* < 0.001) and a weak correlation between urinary CTX-II levels and WOMAC index scores (r = 0.367, *p* < 0.001). Multiple regression analysis showed that urinary CTX-II was independently associated with KL grades. Whereas age, gender, and WOMAC index had no statistically significant influence on the urinary CTX-II levels.

**Conclusions:**

Patients with knee OA had higher urinary CTX-II levels than healthy controls. Moreover, levels of urinary CTX-II were independently correlated with radiographic severity of knee OA. Age, gender, and patient-reported outcomes exerted no effect on the urinary CTX-II levels.

**Level of evidence:**

Diagnostic Level III.

## Background

Osteoarthritis (OA) represents the most common chronic joint disease that contributes to joint pain and disability. OA has a negative impact on the physical and mental health of patients who suffer from the condition. The prevalence of OA has been on the rise. Data from the National Health Interview Survey estimated that 14 million people in the US have symptomatic knee OA [1, 2]. Moreover, more than half of patients suffering from knee OA are under the age of 65 years. OA has recently been described as a whole-joint disease that involves the degradation of the articular cartilage, thickening of the subchondral bone, synovial inflammation, degeneration of ligaments, and hypertrophy of the joint capsule. The disease arises due to an imbalance between the repair and destruction of joint tissues [3]. Early diagnosis of knee OA remains a diagnostic challenge due to the limited signs and symptoms presented during the early phase of the disease. Radiographic evidence of OA is a potential late sign that irreversible joint damage may have already occurred [4]. Magnetic resonance imaging (MRI) and biomarkers are potential diagnostic tools used to overcome this problem. However, the use of MRI is limited by cost, availability, and the absence of a validated international scoring system [5].

Several studies have evaluated the role of various diagnostic biomarkers in knee OA. The biomarkers of cartilage degradation compared to other biomarker categories have been extensively investigated as degradation of articular cartilage is considered to be a central feature of OA [6–9]. C-terminal cross-linked telopeptide of type II collagen (CTX-II) is a byproduct of type II collagen breakdown that is localized almost exclusively in cartilage [10]. Previous studies have demonstrated that urinary CTX-II levels are significantly elevated in patients with knee OA compared to that in healthy controls [11–13]. Additionally, recent meta-analyses showed the potential of using urinary CTX-II levels to distinguish between patients with knee OA from healthy controls. However, this diagnostic feature is not upheld in Asian populations, probably due to the small number of studies that were conducted in Asia [14–16].

Therefore, the aims of this study were to evaluate the difference in urinary CTX-II levels between patients with knee OA and healthy controls, to determine the correlation between urinary CTX-II levels, radiographic severity of OA, and patient-reported outcomes and to evaluate the effect of age and gender on urinary CTX-II levels in the Asian populations.

## Methods

### Participants

This prospective cross-sectional study included 78 patients with knee OA aged > 40 years, who satisfied the diagnostic criteria for knee OA described by the American College of Rheumatology (ACR) [17], from December 2018 to June 2019 at Phyathai 2 hospital. There were 18 males and 60 females, with ages ranging between 41 and 85 years. The average (± standard deviation) age was 64.45 (± 10.66) years. The exclusion criteria included the presence of secondary knee OA, chronic kidney disease [glomerular filtration rate (GFR) < 30 ml/min], OA of other joints such as hip, hand, and spine, current or previous OA treatment such as intraarticular hyaluronic and steroid injection, symptomatic slow-acting drugs for osteoarthritis (SYSADOAs) such as diacerein and glucosamine sulfate within the last 3 months, and current or previous treatment with bisphosphonate [18]. The control group consisted of 51 healthy participants aged > 40 years without clinical or radiographic evidence of knee OA as indicated by a Kellgren and Lawrence (KL) grade 0 or 1 [19]. There were 20 males and 31 females in this group and their ages ranged from 40 to 73 years, with an average age of 51.53 (± 7.54) years.

Baseline characteristics, including age, gender, height, weight, and body mass index (BMI), were collected. Radiographic severity of the OA, Western Ontario and McMaster (WOMAC) index scores, and urinary CTX-II levels were evaluated in all participants. The study was approved by Phyathai 2 hospital ethics committee and all participants provided written informed consent.

### Radiographs

Bilateral knee radiographs were taken in a weight-bearing anteroposterior view. Two orthopedic surgeons independently assessed the severity of the tibiofemoral OA according to the KL grading system by qualitatively evaluating the presence of osteophytes, joint space narrowing, and subchondral bone sclerosis [19]. Whenever there was a difference between left and right knees, the higher KL grade was included in the analysis. OA was defined as the presence of at least 1 knee with a KL grade 2 or higher. Whenever the KL score assigned by the orthopedic surgeons differed, the 2 readers met to read the radiographs together, and a consensus score was determined.

### Urinary CTX-II levels

A morning urine sample (5–10 ml) was collected and stored at 4 °C until the analysis was performed on the same day. Urinary CTX-II was measured using a competitive ELISA test (CartiLaps; Nordic Bioscience, Herlev, Denmark). This assay uses a monoclonal antibody, mAbF46, specific for a 6-amino-acid epitope (EKGPDP) derived from the collagen type II C-terminal cross-linked telopeptide [10]. CTX-II levels were corrected for by urinary creatinine concentration using the following formula: corrected CTX-II value (ng/mmol Cr) = 1000 x urine CartiLaps (μg/L) / creatinine (mmol/L).

### Patient-reported outcome measurement

The WOMAC index is a multidimensional, self-reported questionnaire for patients with OA of the hip or knee [20]. It is composed of 24 questions, covering categories on pain (5 items), stiffness (2 items), and function (17 items). The WOMAC total score is determined by an average score of all dimensions (0–10 points) [20, 21].

### Statistical analysis

The sample size was calculated to detect a difference in urinary CTX-II levels between groups according to the previous study [11]. Using a significance level of 0.05 with a power of 80%, the sample was estimated at 50 subjects for each group. IBM SPSS Statistics for Windows, version 20 (IBM Corp., Armonk, N.Y., USA) was used to perform the statistical analysis. Measured data were expressed as mean ± standard deviation. Independent sample *t*-tests were used to evaluate statistical differences between the 2 groups. One-way analysis of variance (ANOVA) was used to analyze the statistical differences among multiple groups. Correlation analysis was performed using Pearson’s correlation coefficient. Correlations were classified as very weak [correlation coefficient (r) < 0.20), weak (r = 0.20–0.39), moderate (r = 0.40–0.59), strong (r = 0.60–0.79), or very strong (r > 0.80). Multiple linear regression analysis was also conducted. *P* values less than 0.05 were considered statistically significant.

## Results

The study included 78 patients with knee OA, consisting of 25 patients with KL grade 2, 26 patients with KL grade 3, and 27 patients with KL grade 4. Mean age in this group was 64.45 ± 10.66 years. The total WOMAC score ranged from 0.07 to 7.96 with a mean score of 3.74 ± 2.12. The control group consisted of 51 participants with a mean age of 51.53 ± 7.54 years. The mean age in the OA group was higher than that in the control group. There was no statistically significant difference in sex, height, weight, or BMI between the 2 groups (Table 1).

**Table 1:** Demographic data

Characteristics	Control (*n* = 51)	OA (*n* = 78)			
			KL 2 (*n* = 25)	KL 3 (*n* = 26)	KL 4 (*n* = 27)
Age (years)	51.53 ± 7.54	64.45 ± 10.66	58.24 ± 9.73	64.62 ± 10.21	70.04 ± 8.91
Gender, Female (*n*, %)	31 (60.78)	60 (76.92)	21 (84)	21 (80.76)	18 (66.66)
Height (cm)	161.21 ± 6.47	158.67 ± 7.9	160.2 ± 6.72	159.03 ± 7.57	156.9 ± 9.07
Weight (kg)	65.97 ± 9.41	68.05 ± 14.51	64.27 ± 11.05	65.86 ± 10.15	73.67 ± 18.99
BMI (kg/m^2^)	25.36 ± 3.19	26.97 ± 4.94	25.07 ± 4.27	26.07 ± 3.97	29.59 ± 5.37
WOMAC index	1.14 ± 1.72	3.73 ± 2.12	1.98 ± 1.74	3.63 ± 2.01	5.23 ± 1.29
CTX-II (ng/mmol Cr)	178.27 ± 125.39	418.23 ± 392.87	322.84 ± 269.4	387.54 ± 319.9	536.11 ± 517.3

### Comparison of urinary CTX-II levels between knee OA patients and healthy controls

**Fig. 1:**
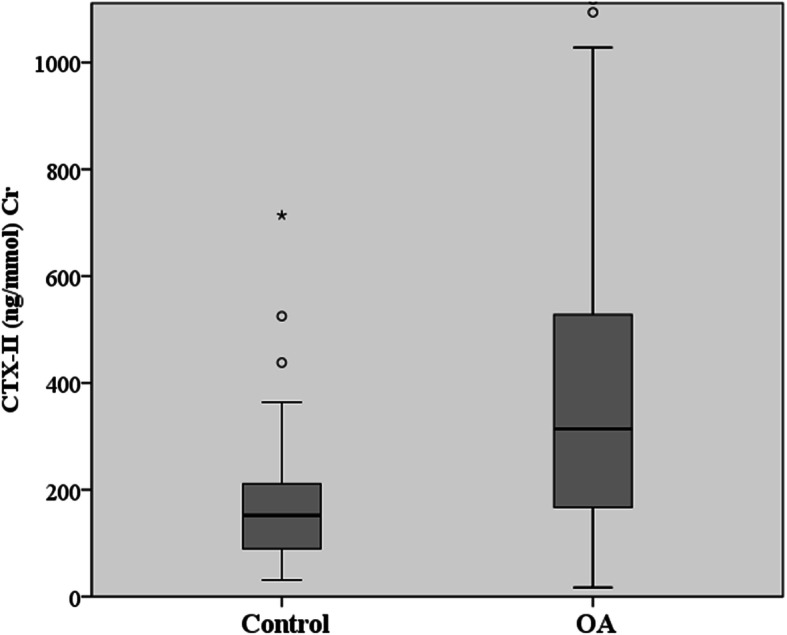
Comparison of the levels of urinary CTX-II between knee OA patients and controls

Mean concentration of urinary CTX-II in the OA group was 418.23 ± 392.87 ng/mmol Cr. Mean concentration of urinary CTX-II in the control group was 178.27 ± 125.39 ng/mmol Cr. Urinary CTX-II levels were significantly higher in the OA group than in the control group (*p*< 0.001) (Fig. 1).

### Correlation between urinary CTX-II levels and different radiographic categories of OA severity

**Fig. 2:**
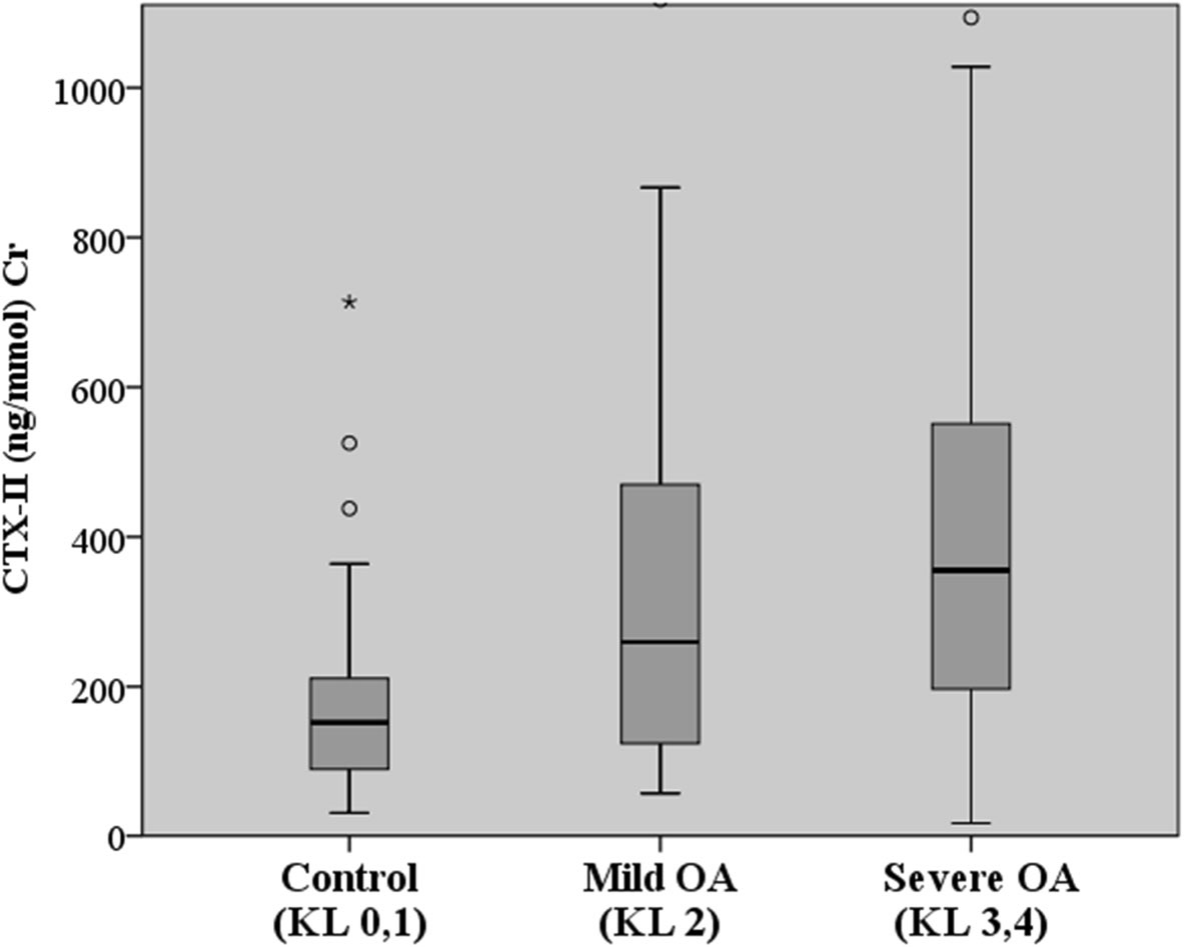
Comparison of the levels of urinary CTX-II in different radiographic knee severities

Patients with radiographic KL grade 2 were classified as having mild knee OA, and patients with radiographic KL grade 3 and 4 were classified as having severe knee OA. According to the results, the severe knee OA group had significantly higher urinary CTX-II levels than that in the control group with levels of 463.23 ± 434.31 ng/mmol Cr and 178.27 ± 125.39 ng/mmol Cr, respectively (*p* <0.001). However, the results were not significantly different between the mild OA (322.84 ± 269.4 ng/mmol Cr) and control groups (*p* = 0.182) and between the severe and mild OA groups (*p* = 0.2) (Fig. 2).

### Correlation between urinary CTX-II levels, radiographic knee OA severity, and patient-reported outcomes

A statistically significant positive correlation was found between urinary CTX-II levels and KL grades (r = 0.405, *p* < 0.001). There was a positive correlation between urinary CTX-II levels and WOMAC index (r = 0.367, *p*< 0.001). No correlation could be found between urinary CTX-II levels and weight, height, or BMI (Table 2).

**Table 2:** Correlation between urinary CTX-II and the parameters

Parameter	Urinary CTX-II	
	r	*P*-value
Age	0.160	0.07
BMI	0.167	0.058
WOMAC index	0.367	< 0.001*
Kellgren and Lawrence	0.405	< 0.001*

Furthermore, we investigated the possible relation between urinary CTX-II and the parameters using multiple linear regression analysis. It showed that urinary CTX-II was independently associated with KL grades, with the standardized regression coefficient being 0.440. Whereas age, sex, BMI, and WOMAC index exerted no statistically significant influence on the urinary CTX-II levels (Table 3).

**Table 3:** The link between urinary CTX-II and the parameters as assessed by multiple linear regression analysis

Parameters	B	SE	Beta	*t*	*P*-value
(Constant)	82.893	292.001		0.284	0.777
Age	−4.866	3.407	−0.165	−1.428	0.156
Sex	75.495	65.496	0.099	1.153	0.252
BMI	2.585	6.970	0.034	0.371	0.711
WOMAC index	20.279	16.967	0.139	1.195	0.235
Kellgren and Lawrence	126.544	39.891	0.440	3.172	0.002*

### Subgroup analysis

We divided the participants into 2 age categories, namely, age 40–59 years and age ≥ 60 years groups. In the control group, there was no statistically significant difference in urinary CTX-II levels between the 2 age categories with levels of 181.61 ± 133.28 ng/mmol Cr and 162.66 ± 83.06 ng/mmol Cr, respectively (*P* = 0.685). There was also no significant correlation between urinary CTX-II levels and the age of the patients in the control group (r = 0.144, *P* = 0.313).

In the OA group, we evaluated the relationship between urinary CTX-II levels and the 2 age categories in the mild and severe OA groups, independently. There was no statistically significant difference in urinary CTX-II levels between the 2 age categories in both mild OA and severe OA groups (*p* = 0.563 and *p* = 0.198, respectively). There was no significant correlation either between urinary CTX-II levels and age in both mild and severe OA groups (r = − 0.243, *P* = 0.241 and r = − 0.135, *P* = 0.333, respectively).

Comparison between genders showed that there was no statistically significant difference in urinary CTX-II levels between healthy males and females, with the levels being 181.45 ± 108.93 and 176.22 ± 136.67 ng/mmol Cr, respectively (*p* = 0.886). In the severe OA group, mean urinary CTX-II levels in females were higher than that in males, but this difference was not statistically significant, with the levels being 511.92 ± 486.21 ng/mmol Cr and 327.57 ± 191.13 ng/mmol Cr, respectively (*p* = 0.175). A statistical analysis of the mild OA group was not performed because of the small number of male subjects in this group.

There was a significant correlation between urinary CTX-II levels and age in the healthy female control group (r = 0.403, *P* = 0.025) but not in the healthy male control group or the OA group.

### Potential confounders

In the severe OA group, 14 patients underwent unilateral total knee arthroplasty (TKA) at least 6 months prior to urinary collection. When urinary CTX-II levels were compared between patients who had undergone unilateral TKA and those who had not (non-TKA) in the severe OA group, higher levels were found in the non-TKA group, but this was not statistically significant with the levels being 515.74 ± 472.26 ng/mmol Cr and 316.93 ± 266.91 ng/mmol Cr, respectively (*P* = 0.143).

## Discussion

In recent years, biomarkers of OA have become a particular area of interest. The role of each biomarker was evaluated in relation to OA diagnosis, burden of disease, OA progression, and efficacy of interventions [9, 22]. The main objectives of biomarker research were to identify the disease in the earlier stages, to stratify those at risk of disease progression, and to enable the innovation of new interventions. Several biomarkers in the categories of cartilage synthesis, cartilage degradation, bone synthesis, bone degradation, synovial tissue synthesis/anabolic activity, and synovial tissue degradation/catabolic activity were evaluated by several studies. In our study, we focused on the biomarkers of cartilage degradation, since it is one of the main pathologies of OA. According to systematic reviews, urinary CTX-II is the most effective cartilage degradation biomarker in most aspects in terms of diagnosis, severity of disease, and prognosis [5, 9, 16].

In this study, we found that the urinary CTX-II levels in patients with knee OA were higher than that in healthy controls (*p* < 0.001). This finding was in accordance with the result of previous studies [12, 13, 23]. Therefore, urinary CTX-II levels can be used as a diagnostic marker of knee OA. One of the problems with using urinary CTX-II as a diagnostic biomarker for knee OA is the variation in the optimum cutoff level. A recent meta-analysis showed that mean urinary CTX-II levels ranged between 129 and 345 ng/mmol Cr in the healthy population [14, 16]. Moreover, factors that influence CTX-II levels remain unclear.

This study demonstrated that the increase in urinary CTX-II levels was associated with an increase in radiographic OA severity. Although the difference was not statistically significant, the severe OA group had higher urinary CTX-II levels than that in the mild OA and control groups. Furthermore, a moderate correlation was found between urinary CTX-II levels and KL grades (r = 0.405, *p* < 0.001) and the multiple linear regression analysis demonstrated that urinary CTX-II was independently associated with KL grades, suggesting that urinary CTX-II levels are increased in patients with an increased OA severity. Recent meta-analysis also revealed that urinary CTX-II levels were consistently increased in patients with severe knee OA on radiographic evaluation compared to that in those with mild knee OA [14, 15, 24]. Therefore, urinary CTX-II could be a useful tool for monitoring OA disease progression and treatment outcomes.

The correlation between urinary CTX-II levels and patient-reported outcome scores was discussed in previous studies [25–27]. In many published studies, it has been shown that urinary CTX-II levels were not related to patient-reported outcome scores [25, 27]. However, a single study has shown a strong correlation [26]. In the current study, we used WOMAC index since it is simple and quick to administer and score against guidelines provided [28]. The population’s language version of WOMAC index also had a good validity and reliability [21]. We found a significant but weak correlation between urinary CTX-II levels and WOMAC index scores (r = 0.367, *p* < 0.001). Nonetheless, multiple regression analysis showed that no independent association existed between urinary CTX-II levels and WOMAC index. This might be caused by the fact that WOMAC index increases with increase in KL grades.

The influence of age and gender on urinary CTX-II levels was evaluated in previous studies [14, 29–32]. In this study, the multiple regression analysis revealed that that age and gender had no statistically significant influence on the urinary CTX-II levels. However, the subgroup analysis exhibited a significant increase in urinary CTX-II levels with increase in age in the healthy female control group. Our results are consistent with the findings of a previous study [32]. One of the reasons that urinary CTX-II levels are elevated in the female population might be ascribed to the effect of menopause [29, 32–34]. However, some studies showed slight rise in urinary CTX-II concentration with increase in age in those older than 55 years in both genders. Nevertheless, the higher concentration seems to reflect that the prevalence of radiographic OA increases with increase in age [29, 30].

This study had potential limitations. First, the study used plain radiographic measurement to diagnose and assess OA severity and the method may not have as great a sensitivity and specificity as MRI. Nonetheless, X-ray is still the most widely used imaging modality in clinical practice, rendering application of the results more suitable. Second, this study was of a cross-sectional design, and CTX-II was only measured at a single point of time. Thus, we could not investigate whether urinary CTX-II levels might be predictive of incidence and disease progression of OA from this study. Third, some patients underwent unilateral TKA surgery that might affect the urinary CTX-II levels and WOMAC index. Fourth, we did not have radiographs of the other asymptomatic joints. Additionally, we used KL grade 2 as the generally accepted cutoff point to define knee OA radiographically, although, KL grade 0 and 1 may represent different entities [35]. Finally, the sample size of the study was relatively small when stratifying results into each KL grade.

## Conclusions

This study showed that the urinary CTX-II levels in patients with knee OA were significantly higher than in healthy controls. Therefore, urinary CTX-II levels can be used to distinguish patients with knee OA from control subjects. Moreover, levels of urinary CTX-II were independently correlated with radiographic severity of knee OA. Thus, it can be used as a disease monitoring tool. Age, gender, and patient-reported outcomes had no effect on the urinary CTX-II levels. Further longitudinal studies with larger sample sizes are warranted to evaluate the combination of several biomarker categories and the efficacy of each treatment.

## Data Availability

The datasets used during the current study are available from the corresponding author on reasonable request.

## Abbreviations

ACR: American College of Rheumatology
BMI: Body mass index
CTX-II: C-terminal cross-linked telopeptide of type II collagen
GFR: Glomerular filtration rate
KL: Kellgren-Lawrence grading
MRI: Magnetic resonance imaging
OA: Osteoarthritis
TKA: Total knee arthroplasty
WOMAC: Western Ontario and McMaster
